# Multiple Sources of Riparian Wetland Suspended Solids during Episodic Rain Events: Influence on Uranium Transport

**DOI:** 10.1021/acs.est.5c08896

**Published:** 2025-10-22

**Authors:** Daniel I. Kaplan, Karah M. Greene, Wei Xing, Brian A. Powell, Maxim I. Boyanov, Edward J. O’Loughlin, Kenneth M. Kemner, Arelis M. Rivera-Giboyeaux, Peng Lin

**Affiliations:** † Savannah River Ecology Laboratory, 1355 University of Georgia, Aiken, South Carolina 29808, United States; ‡ Department of Environmental Engineering & Earth Sciences, 2545 Clemson University, Anderson, South Carolina 29634, United States; § Biosciences Division, 1291 Argonne National Laboratory, Lemont, Illinois 60439, United States; ∥ Institute of Chemical Engineering, Bulgarian Academy of Sciences, Sofia 1113, Bulgaria; ⊥ Atmospheric Technologies Group, 1073 Savannah River National Laboratory, Aiken, South Carolina 29808, United States

**Keywords:** iron flocs, natural organic matter, rainfall, stream flow rate

## Abstract

Suspended solids can be the primary vector for transporting contaminants in streams. The objective of this study was to determine whether changes in the properties of suspended solids during rain events impacted contaminant transport. Stream water was collected during five episodic events downstream from a U-contaminated wetland located in South Carolina, USA. The suspended particles were initially composed of Fe-flocs (particles formed *in situ* prior to the rain event) that had significantly greater Fe, Mn, organic-C, and U content than particles collected later during a sampling rain event. XANES and EXAFS revealed that U in the Fe-flocs was U­(VI) and that it was not incorporated in a mineral structure but existed as inner- or outer-sphere adsorbed uranyl species associated with organic matter and Fe-oxides. The uranyl had an extraordinarily high affinity for the suspended solids, with solid to liquid U ratios of >72,000 (μg/kg)/(μg/L). After the initial flush of Fe-flocs, a greater fraction of the suspended solids had lower organic-C, Fe, Mn, and amorphous phases and were composed of more quartz, kaolinite, and gibbsite, resulting in lower U concentrations than those in the solids collected earlier in the rain event. This study highlights the importance of understanding suspended solids as transport vectors and their potential dynamic nature during rain events.

## Graphical abstract

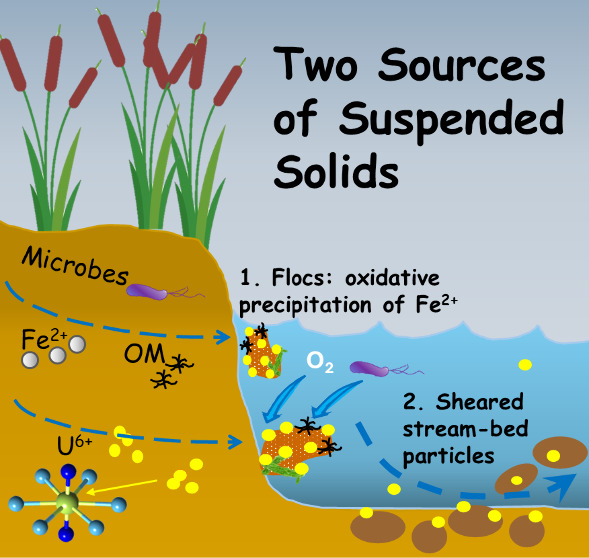

## Introduction

Suspended solids play an important role in transporting contaminants in streams.^1–3^ Concentrations of only a few milligrams per liter of stream water often account for >90% of the system’s contaminant load.^2^ For this reason, particular attention in modeling stream contaminant transport is directed at quantifying the affinity of the contaminant to the suspended solids and the impact of flow rate on the generation of suspended solids.^1^ In turn, there are several meteorological, topographical, geochemical, biological, and anthropogenic factors that directly or indirectly influence these first-order modeling parameters (reviewed by Bilotta and Brazier).^2^

Understanding contaminant transport in streams running through wetlands is especially important because these landforms are effective at attenuating contaminants due to their unique hydrological and biogeochemical properties.^4^ Hydric conditions promote vegetative growth, which in turn enriches the sediment with organic matter and microbes. Furthermore, fluctuations in sediment moisture content can create a gradient of the pH and redox potential. Together, these wetland conditions create diverse biogeochemical environments that promote the attenuation of many contaminants entering the wetlands from surface waters or subsurface aquifers, leading to wetlands often being described as “kidneys of the landscape”.^5^

Sources of suspended solids from wetlands are especially diverse. Suspended solids of large rivers, especially those that are channelized, originate primarily from the shearing of particles from the river floor and the riverbanks. Should the banks of rivers or their tributaries overflow, the newly flooded lands also contribute suspended solids to the rivers. Suspended solids from wetlands not only originate from stream floors and stream banks, but they may also arise from flocs.^6^ These particles are formed *in situ* in hydrologically gaining streams, that is, streams receiving a fraction of their water from the adjoining land, as opposed to hydrologically losing streams that lose water from the stream to the adjoining land. In gaining regions of a riparian wetland, the subsurface porewater often contains dissolved ferrous ions that flow into the oxygenated stream and precipitates as Fe-oxyhydroxide. These iron flocs are composed of elevated levels of ferrihydrite, lepidocrocite, organic matter, and microbes.^7–9^ When the hydrological and biogeochemical conditions are favorable and stream flow is slow, the flocs start accumulating at the air–water–sediment interface and then continue forming along the stream floor. High-flow conditions during rain events transport the flocs downstream, leaving the water clear again, with flocs reappearing once stream flow returns to the baseline.

The objectives of this study were to build on the results of Batson et al.,^10^ who reported that most stream water U in Tims Branch was associated with suspended solids, and determine whether the geochemical properties of suspended solids change during rain events and, if so, determine how these changes impact contaminant transport. Our approach was to collect stream water during five episodic rain events downstream from a U-contaminated wetland located in South Carolina, USA, and then compare the suspended solids’ geochemical properties to U concentrations before, during, and after the rain events.

## Materials and Methods

### Study Site

The study site was the Tims Branch wetland located on the Savannah River Site, a nuclear material production facility in Aiken, South Carolina, USA (Figure 1; Reed and Swanson).^11^ The wetlands received waste released from a nuclear fuel fabrication facility during the 1970s. Included in the waste was 43,500 kg of depleted U. A recent land survey involving >700,000 gamma spectra revealed that approximately 94% of the released U remains immobilized in five multihectare hot spots, with sediment U concentrations as high as 14,000 mg/kg.^12,13^ Hydrological watershed modeling indicated that the location of the five hot spots coincided with areas where Tims Branch flow rate was slow.^13^ Suspended solids are the primary vector for transporting U,^10^ and presumably, these particles would have greater tendency to settle in areas where the flow was slow. Sediment cores revealed that most of the sediment U was located either in the upper 4 cm or buried in a layer at a 4 to 8 cm depth. X-ray absorption near edge structure (XANES) and extended X-ray absorption fine structure (EXAFS) measurements indicated that the U existed primarily as U­(VI), except in water-saturated deeper layers where U­(IV) predominated. The U­(VI) existed primarily as uranyl, UO_2_^2+^, that was mainly associated with ferrihydrite and to a lesser extent natural organic matter.^14^ Fourier-transform ion cyclotron resonance–mass spectrometry analysis of sediment organic matter from the wetland identified 9614 different organic matter formulas, of which 715 formulas contained U. The U containing formulas tended to be enriched with Fe, N, and/or S and were composed primarily of lignin-like and protein-like molecules.^8^

**Figure 1:**
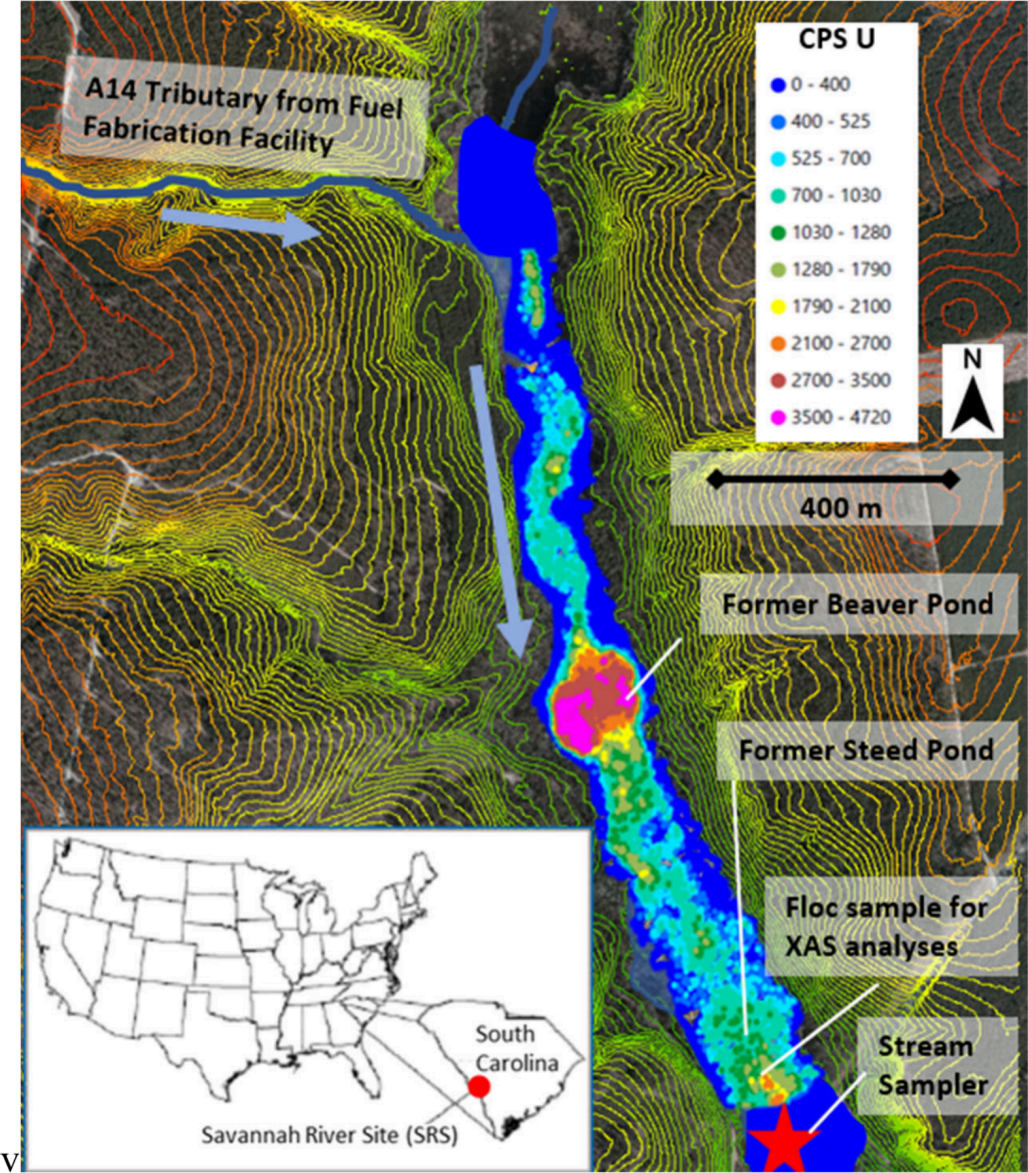
LIDAR topography and sediment U activity (counts/s) at the Tims Branch Study Site (sediment U activity was previously reported).^12^

The stream water sampling location was 50 m downstream from the two most contaminated hot spots (Figure 1; photographs of the location are provided in Figure S1). The robotic sampler (Model 6712; Teledyne ISCO) was equipped with an area velocity flow module that is based on Doppler technology (750 Area Velocity Flow Module, Teledyne ISCO). Nominally, the sampler was programed to collect 1 L samples every 1 or 1.5 h, where each sample was an aggregate of four 0.25 L subsamples evenly distributed during the sampling period. As such, each aggregated sample was designed to represent the average of each sampling interval. The day after each of the five sampling events, each 1 L polypropylene sample bottle was individually checked to ensure they did not exceed radiological limits, placed on ice, and transported 5 km to a 5 °C refrigerator for storage. In the lab, subsamples of all stream water samples were passed through 0.45 μm pore size polyethersulfone (PES) filters to collect suspended solids (>0.45 μm) for solid phase characterization and the filtrate samples (<0.45 μm) for aqueous phase characterization. Initial studies indicated that a colloidal fraction, operationally defined as <0.45 μm and >3000 molecular weight cut off, did not contain a significant fraction of U.^15^ The concentration of total suspended solids (TSS) was determined by the dried weight difference between the preweighed filter and the filter with solids, divided by the volumes of the stream water filtered. The filtrate samples were stabilized in 2% HNO_3_ until they were chemically analyzed.

A one-time stream sampling for Fe-flocs was conducted during base-flow conditions from Steed Pond, located about 70 m upstream from the stream sampler (Figure 1). These samples were collected by dipping a 50 mL centrifuge tube into the stream and collecting water at >5 cm from the water–air interface and >5 cm from the stream floor. The samples were collected in December 2022 to coincide with available beam time at the Advanced Photon Source, Lemont, Illinois, USA. As such, they are intended to represent base-flow in a portion of the stream that has gaining hydrological conditions. This portion of Tims Branch has gaining hydrological conditions for 67% of the year.^13^

Hourly rainfall data were obtained by using the Multi-Radar Multi-Sensor (MRMS) system^16^ developed by the NOAA National Severe Storms Laboratory (NSSL). This system estimates rainfall for a given location by combining data from various sources, including approximately 180 radar stations across the USA and Canada, satellite data, remote sensing, lightning, rain gauge observations, and other environmental information. The MRMS system was previously validated for the Savannah River Site (SRS) by comparing MRMS rainfall estimates to three tipping bucket gauges for 23 rain events over the course of one year.^17^ Statistical analysis from these comparisons showed a good agreement between MRMS-derived estimates and ground measurements.

### Chemical Analysis

#### Aqueous Phase Characterization

Total U, Fe, and Mn in the <0.45 μm fraction were analyzed in a 2% HNO_3_ matrix via inductively coupled plasma–mass spectrometry (ICP-MS; Thermo X-Series II). In addition to a water blank, appropriate NIST SRM 3100 series single-element standards were carried through the ICP-MS analyses. All chemical analyses were conducted in duplicate.

#### Suspension Characterization

In the lab, well-mixed stream samples were analyzed for turbidity using an Oakton Instruments T100WL turbidity meter. Electrophoretic mobility and particle size distribution from two sampling events were determined by using five replicates by photon correlation spectroscopy (Nanobrook 90PlusPALS, Brookhaven Instruments). Both measurements were conducted with a temperature-controlled 640.0 nm wavelength laser. The Brookhaven Polystyrene Latex Standard and the Brookhaven BI-ZR5 Zeta Potential Reference Standard were run after approximately every 30 sample measurements to ensure consistency. Preliminary studies indicated that the suspended solids aggregated during storage and that mildly sonicating them in a 150 W water bath for 5 min was the minimum duration required to obtain results with consistent size and minimum polydispersity.

#### Solid Phase Characterization

Total U, Fe, and Mn contents of the suspended solids collected on the filters (>0.45 μm) were determined by digesting the solids in concentrated HNO_3_ (heated to 95 °C) and 30% H_2_O_2_ per the EPA 3050B method.^18^ An aliquot of the digestate was analyzed in a 2% HNO_3_ matrix via ICP-MS. In addition to a water blank, the NIST sediment standard no. 8704 was carried through the digestion and ICP-MS analyses. This Buffalo River sediment standard was selected because we were interested in stream sediments and it is certified for all the key elements of interest in this study, including Fe, Mn, Ni, and U.

Randomly oriented powder mounts of ∼0.25 g of suspended solids were prepared for X-ray diffraction (XRD; Bruker D2 PHASER) analysis using standard methods.^19^ XRD patterns of the samples were collected from 3° to 75° 2θ with a 0.02° 2θ step size, integration time of 0.5 s per step, and 30 rpm for the sample rotation speed. Phase identification was conducted by using the Bruker DIFFRAC.EVA software (V7) and the PDF-4+ database (2023) compiled by the International Center for Diffraction Data (ICDD). Phase proportions were determined via Rietveld analysis using Bruker DIFFRAC.TOPAS. Samples were mixed with the National Institute of Standards and Technology (NIST) Silicon Standard Reference Material 640f to enable the determination of amorphous phase proportions. Total particulate carbon and nitrogen in the suspended solids were determined by combustion/IR detection (LECO CNS-2000 analyzer, St. Joseph, Missouri, USA). Because the suspended solids in the study area are typically from acidic sediments (pH 4.25 to 5.37, with an average of 4.63 ± 0.41, *n* = 6),^20^ they contain negligible amounts of inorganic carbon. As such, these parameters can be considered as measures of particulate organic carbon (POC) and particulate nitrogen (PN).

The stream water samples containing flocs (see the location in Figure 1) were shipped overnight on ice to Argonne National Laboratory for X-ray absorption spectroscopy (XAS) analysis. Within 24 h of receipt, ∼10 mL of floc suspension was passed through a 0.2 μm membrane. The filter was cut into several pieces, which were stacked onto a drilled plastic slide and then sealed with Kapton film. The samples were collected from an oxygenated stream, and for this reason, no precautions were taken to minimize air contact with the sample during collection, shipping, or sample mounting.

The samples were analyzed for U speciation at the MRCAT/EnviroCAT Insertion Device beamline (Sector 10, Advanced Photon Source).^21^ XANES and EXAFS spectra were collected at the U L_III_-edge (17,166 eV) from the filtered solids in fluorescence mode using a four-element Vortex detector. Various standards measured previously at the same beamline were used in the analysis, including U­(VI) phosphate,^22^ U­(VI) adsorbed to clays and goethite,^23^ and aqueous U­(VI) or U­(VI) adsorbed on carboxyl functionalized beads.^24^ Nanoparticulate uraninite^23^ was used as a standard for U­(IV). Energy calibration was established by setting the inflection point in the spectrum from hydrogen uranyl phosphate to 17,166 eV and maintained by concurrent collection of data from the sample and standard. Spectra were recorded at −150 °C from six locations on each sample. Radiation-induced shifts in the spectra were not detected.

Fe K-edge (7112 eV) XANES and EXAFS measurements were carried out at the MRCAT/EnviroCAT Bending Magnet beamline (Sector 10, Advanced Photon Source).^25^ Samples were prepared in the same way as for the U measurements except that only a single layer of the filter membrane was used. Spectra were collected at room temperature in quick-scan transmission mode. Energy calibration was established by setting the inflection point in the spectrum of Fe metal to 7112 eV. Radiation-induced changes or sample inhomogeneity was monitored by taking 2 to 3 consecutive spectra at six locations. No differences were observed, so all scans from each sample were averaged to produce the final spectrum. The Fe standards used in the analysis were measured at the same beamline in this and previous work^26^ and include spectra from mackinawite, vivianite, siderite, magnetite, goethite, ferrihydrite, hematite, goethite, and lepidocrocite. Oxidized Fe in clay minerals was represented by the following standards: nontronite, illite, and montmorillonite. Fe­(III) bound to organic matter ligands was represented by powder and solution Fe­(III)-citrate standards. The polycrystalline Fe powders were mounted on the adhesive side of Kapton tape, and their absorption spectra were measured in transmission. The clay minerals were measured as wet pastes mounted in 1.5 mm thick holders in transmission and/or fluorescence mode depending on Fe content.

Normalization and background removal of the Fe and U XAS data were done using the program AUTOBK.^27^ Linear combination fits of the data were performed using the program ATHENA.^28^

## Results and Discussion

### Flow Rate, Turbidity, Suspended Solids, and U Transport

Rainfall during the five sampling events ranged from 3.35 to 9.87 cm and lasted between 9 and 40 h (Table 1). Not surprisingly, most of the U in the stream was associated with suspended solids, U_>0.45 μm_, often accounting for >90% of the U in the stream (U_Total‑Susp_ = U_>0.45 μm_ + U_<0.45 μm_). Conversely, U in the <0.45 μm fraction, U_<0.45 μm_, was consistently below the USEPA maximum contaminant level (MCL) of 30 μg/L U (Table 1). Flow rate and turbidity were significantly correlated during all 5 sampling events (*p* ≤ 0.01; Table 1). Flow rate was also strongly correlated to U_Total‑Susp_. Consequently, it was not surprising that U_Total‑Susp_ was very strongly correlated to turbidity (Table 1).

**Table 1:** Sampling Event Characteristics, Stream U Properties, and Pearson Correlation Coefficients

						Pearson Correlation Coefficients, *r*		
Sampling Event^a^	Total Rainfall (cm)	Sampling Duration (h)	Maximum % of U with Suspended Solids (vol %)^b^	Maximum U_<0.45 μm_ (μg/L)	Maximum U_Total‑Susp_ ^c^ (μg/L)	Flow Rate vs Turbidity	Flow vs U_Total‑Susp_	U_Total‑Susp_ vs Turbidity
A	3.35	36	94.0	10.7	80.1	0.782**^d^	0.409	0.881**
B	3.38	9	93.0	9.0	60.4	0.853**	0.866**	0.993**
D	2.62	17	92.1	4.1	34.3	0.753**	0.777**	0.991**
E	8.04	40	79.1	13.2	28.3	0.815**	0.685**	0.558**
F	9.87	36	92.9	25.2	118.7	0.731**	0.789**	0.935**

a Sampling event C was omitted from this study because the total rainfall during this event was only 0.69 cm, which created minimal or no measurable changes in flow rate, particle concentration, or U concentration changes.

b (U_>0.45 μm_/U_Total‑Susp_) × 100.

c U_Total‑Susp_ = U_>0.45 μm_ + U_<0.45 μm_.

d Asteriks ** indicate significance of the Pearson correlation coefficient at *p* ≤ 0.01. Events A and E had 22 degrees of freedom (d.f.). Events B, D, and F had 7, 15, and 17 d.f., respectively.

Figure 2 provides rainfall, stream flow, U_>0.45 μm_, and U_<0.45 μm_ over the course of sampling events A and D (similar data plots for sampling events B, E, and F are presented in the Supporting Information, Figure S2). Rainfall during sampling at Tims Branch was sporadic, and the rates varied greatly during a given sampling event (Figure 2). Once a storm started, the flow rate of this small third-order stream tended to gradually increase to a maximum and then gradually decrease. In some cases (events A, E, and F) the first moment, or center of gravity, for the flow rate was 2 to 5 h behind that of the rainfall. For other sampling events (events B and D), the first moment for the flow and rainfall occurred simultaneously. The causes of these temporal differences between maximum rainfall and maximum flow rates are not known. During higher flow conditions, U_>0.45 μm_ increased sharply and varied greatly, whereas U_<0.45 μm_ tended to change in a more gradual and systematic manner during the sampling events, never exceeding the maximum contaminant level (MCL) for U (30 μg/L U) (Figures 2 and S2).

**Figure 2:**
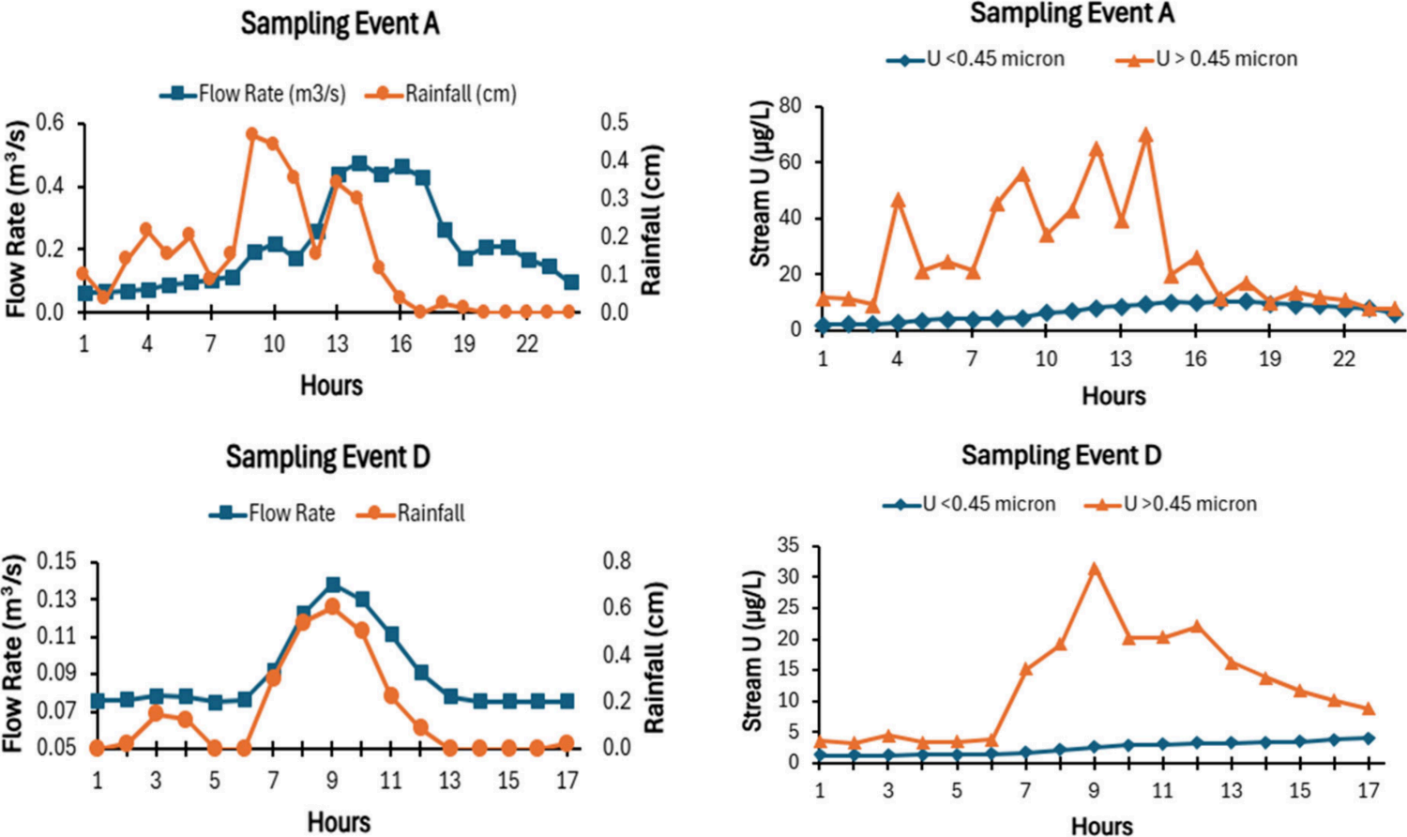
Rainfall, flow rate, U_<0.45 μm_, and U_>0.45 μm_ data collected during sampling events A and D (the Supporting Information provides similar plots for sampling events B–F; Figure S2).

### Suspended Solids Composition and U Concentrations

Uranium concentrations in the suspended solids (U_Solid_) were as high as 968 mg/kg (Table 2). For purposes of comparison, background sediment U concentrations of Tims Branch, based on analyses of samples collected 100 m upstream of the contaminated area, are between 0.5 and 20 mg/kg U.^14^ Conversely, sediment U concentrations from the most contaminated portion of the Tims Branch wetland were as high as 14,000 mg/kg U.^9^ The U concentration ratio U_Solid_/U_<0.45 μm_ with units of (μg U/kg)/(μg U/L) was evaluated to provide a measure of the tendency of the suspended solids to bind U with respect to the aqueous U concentration, a type of partitioning coefficient between the two phases. The suspended sediments had extremely high Log­(U_Solid_/U_<0.45 μm_) values that exceeded 4.87; that is, the U concentrations in the suspended solids were more than 74,000 times greater than those in the aqueous phase (Table 2). Suspended solids U concentrations were very strongly correlated to Fe and Mn concentrations in the suspended solids (Fe_Solid_ and Mn_Solid_, respectively) and had much less or no significant correlations to particulate organic carbon or particulate nitrogen (Table 2). U_Solid_ was not correlated to the total phosphorus concentration in suspended solids (P_Solid_, data not shown).

**Table 2:** Suspended Solids Properties, Including U_Solid_/U_<0.45 μm_ Ratios, U_Solid_, and Pearson Correlation Coefficients between U_Solid_ Concentrations and the Concentrations of Other Chemical Properties

			Pearson Correlation Coefficients, *r*			
Sampling Event	Log(U_Solid_/U_<0.45 μm_)^a^ Ave. ± Stdev. ((μg U/kg)/(μg U/L))	Range of U_Solid_ ^a^ (mg/kg)	U_Solid_–Fe_Solid_	U_Solid_–Mn_Solid_	U_Solid_–POC^b^	U_Solid_–PN^b^
A	4.94 ± 0.30	311–911	0.650**^d^	0.624**	0.188	0.250
B	4.96 ± 0.28	348–608	0.948**	0.787*	0.869**	0.764*
D	5.28 ± 0.22	374–874	0.880**	0.871**	0.596*	–0.562
E	4.69 ± 0.47	120–968	0.960**	0.960**	–0.183	–0.099
F	4.87 ± 0.25	284–577	0.607**	0.638**	0.586**	–0.654
Sediment^c^	3.60 ± 3.14	71–7479	0.529*	--	0.786*	--

a The number of samples per sampling event used to calculate the means and standard deviations are 24 for event A, 9 for event B, 17 for event D, 24 for event E, and 19 for event F. Additional information of U_Solid_ (mg/kg) is presented in the Supporting Information, Figure S5.

b POC = particulate organic carbon; PN = particulate nitrogen. They were measured on suspended solids collected on a 0.45 μm filter.

c Data are from six contaminated sediment samples reported in Lin et al.^20^ that were collected from the former Beaver Pond and Steed Pond (Figure 1).

d Asterisks * and ** indicate significance of the Pearson correlation coefficient at *p* ≤ 0.05 and *p* ≤ 0.01, respectively. All means without an asterisk are nonsignificant at *p* > 0.05.

### U_Solid_ Concentrations Collected Early vs Late during Sampling Events

Tims Branch commonly contains flocs that accumulate in the stream under low-flow base conditions. Images of the sampling site with and without the stream flocs are included in the Supporting Information, Figure S1. Iron flocs recovered from Tims Branch have been shown to have U concentrations as high as 660 mg/kg.^9^ Because the suspended solids appeared quite different within a given time-series sampling event, an evaluation of the suspended solids’ properties collected early vs late in the time series was conducted. It was anticipated that the early samples would include more flocs when compared to the samples collected later in the time series, after the flocs had been transported downstream and flow rates had increased, which would contain fewer flocs and more particles sheared from the stream floor.

Early samples were generally defined as those samples collected prior to the maximum flow rate, while the late samples were those collected afterward (the number of early and late samples for each sampling event are identified in Table 3). For all sampling events, U_Solid_ and the ratio of U_Solid_/U_<0.45 μm_ were significantly greater in the early samples than in the late samples (Table 3). This coincided with significantly greater Fe, Mn, and POC concentrations in the early samples than in the late samples. The distribution of particle diameter sizes, as measured by dynamic light scattering, from two out of the four sampling events analyzed was significantly greater in the early samples than in the late samples. Upon closer inspection and preliminary tests involving sonicating the samples, it was apparent that the early samples were especially prone to forming aggregates. The zeta potential, a measure of electrostatic surface charge, indicated that the early samples had a greater negative change than the late samples for only one of the two events tested. Additional work is needed to determine whether particle size and surface charge are, in fact, distinguishing properties between these two particle categories (Table 3).

**Table 3:** Suspended Solids Characteristics before (Early Samples) and after (Late Samples) Peak Flow during Each Sampling Event

	Event A		Event B		Event D		Event E	
Samples	Early	Late	Early	Late	Early	Late	Early	Late
# of samples^a^	14	9	4	5	8	9	13	11
U (mg/kg)	625**^d^	445	536**	380	541*	432	562***	181
Fe (wt %)	14.81**	8.01	29.74***	7.31	39.74***	16.44	26.77***	4.65
Mn (mg/kg)	1844**	1031	4304***	1315	4970***	2476	3909***	511
POC (mg/kg)	0.065*	0.044	0.112***	0.060	0.105***	0.059	0.050	0.053
PN (mg/kg)	0.0051	0.0041	0.010*	0.0051	0.0076	0.0049	0.0047	0.0049
Log(U_Solid_/U_<0.45 μm_)^b^	5.12***	4.67	5.25***	4.73	5.52***	5.10	4.75***	4.47
Diameter (nm)	267***	215	263	314	247	268	259*	226
Zeta potential (mV)	–19.5	–18.9	NA^c^	NA	NA	NA	–18.2	–20.2***

a Number of samples collected for early or late sample categories (Figures 2 and S2).

b Asterisks *, **, and *** indicate significantly greater value at *p* ≤ 0.05, *p* ≤ 0.01, and *p* ≤ 0.001, respectively, based on single factor ANOVA between early and late samples for a given sampling event.

c U_Solid_/U_<0.45 μm_; units = (μg U/kg)/(μg U/L) = L/kg.

d NA = not analyzed.

Sufficient mass (>0.3 g) of suspended solids collected during sampling events E and F was available to conduct XRD analyses (Table 4; an example of an XRD scan is included in the Supporting Information, Figure S3). Detectable minerals (>∼2 wt %) in the suspended solids included quartz, kaolinite, gibbsite, and various amorphous phases, including Fe-, Al-, and Mn-oxyhydroxides). These phases have been previously identified in the <2 μm fraction of nearby sediments^29^ and in subsurface mobile colloids.^30^ One mineral not identified in the suspended solids but commonly reported in Savannah River Site sediments was hydroxy-interlayered vermiculite. The early samples tended to have greater amorphous phases, whereas the late samples tended to have more quartz. This is consistent with the notion that flocs comprised a larger fraction of the early samples and that stream floor particles tended to comprise more of the late samples.

**Table 4:** Mineralogy of Suspended Solids Based on XRD from Early and Late Samples from Sampling Events E and F^a^

Event	Sample	Amorphous Phases (wt %)	Quartz (wt %)	Kaolinite (wt %)	Gibbsite (wt %)	Goodness of XRD Fit^b^
E	Early	58.4	17.3	22.3	2.0	1.19
	Late	11.7	37.9	43.5	6.8	2.03
F	Early	46.6	20.0	28.4	5.0	1.21
	Late	39.7	30.8	26.1	3.3	1.40

a To collect sufficient mass of suspended solids to do XRD measurements, 4 to 5 individual stream samples were combined from either the early or late samples. An example of an XRD scan is provided in the Supporting Information, Figure S3. There was insufficient suspended solids mass to permit identification and/or quantification of minerals from other sampling events.

b Goodness of fit was estimated using the chi-squared statistic, which tends to 1 as the fit of the experimental data improves.

### XANES and EXAFS Analyses of Floc Samples

The XANES data indicate that 100 ± 5% of the Fe in the flocs existed as Fe­(III) (Figure 3). The spectrum is similar to the ferrihydrite standard; however, differences are noticeable in both the XANES and EXAFS data. Linear combination (LC) fits of the EXAFS data with up to three of the standards described in the Materials and Methods section indicate that 70% of the Fe is in ferrihydrite (Fh), with the remainder distributed between a minor lepidocrocite component (13%) and an Fe­(III) phase that is fit well by the Fe­(III)-citrate solution standard (17%). The latter spectrum represents hydrated Fe­(III) atoms that are loosely bound to an organic ligand, and this standard has been used in previous work as a proxy of Fe­(III) bound to organic matter.^31,32^

**Figure 3:**
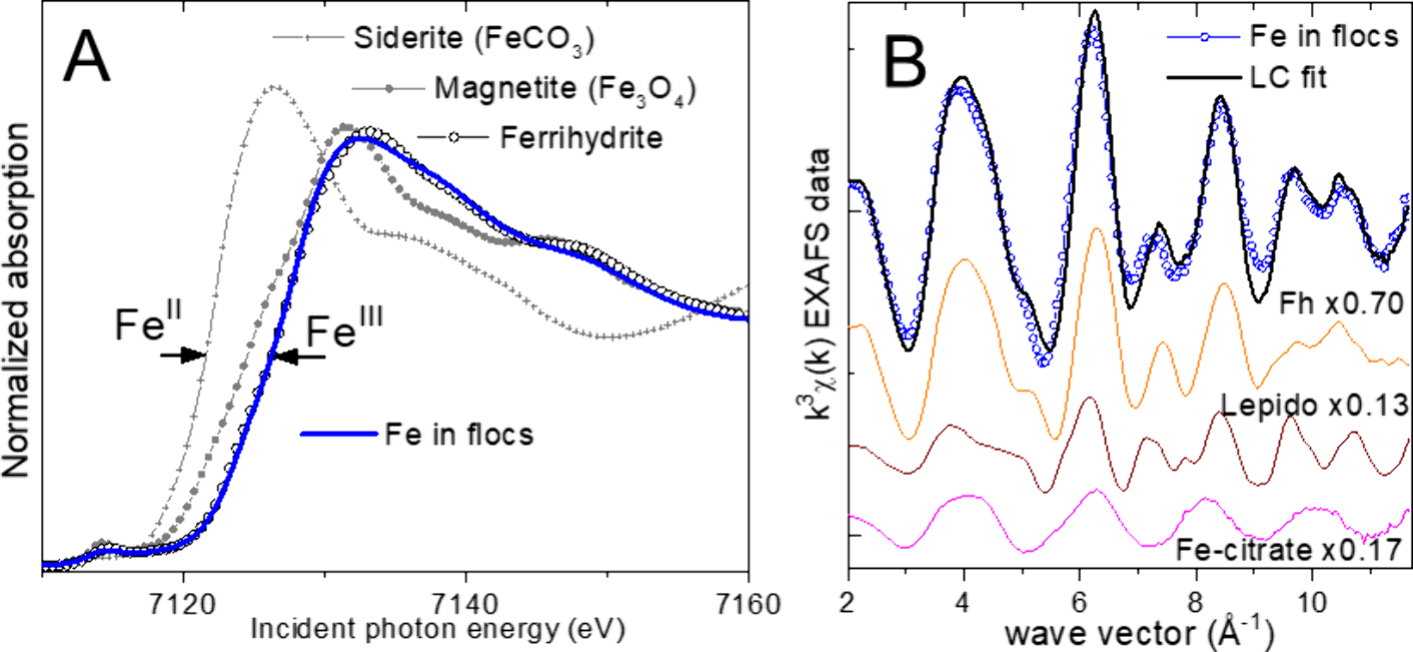
Fe K-edge X-ray absorption spectra from stream flocs. (A) XANES spectrum compared with standards, indicating the predominance of Fe­(III). (B) Linear combination (LC) fit of the EXAFS spectrum. Fh represents ferrihydrite (∼70% of the detected Fe). Lepido represents lepidocrosite (∼13%). Fe-citrate represents Fe­(III) loosely bound to organic carbon (∼17%). The scaled fit components are offset vertically below the data and fit; the refined proportions are shown next to each component (uncertainties are ±5%).

The U L_III_-edge XANES data indicate that U was present as U­(VI) (100 ± 5%) (Figure 4). Because of the high U_Solid_ and low U_<0.45 μm_ concentrations in the stream samples, the spectrum from the filtered solids reflects the speciation of U in the solid phase (i.e., U in the porewater is negligible). Comparison of the EXAFS data to U­(VI) mineral standards did not show a match, indicating that U­(VI) was not in a precipitate but was adsorbed. The major adsorbent groups in the floc system are Fe-oxides and organic matter, so spectra of potential adsorbed U species are compared to the U-floc data in Figure S4. U­(VI) adsorbed to goethite and to SYn-1 clay^23^ were used as proxies of mineral-associated U, and U­(VI) adsorbed to carboxyl functionalized beads^23^ or in U­(VI)-phosphate^22^ were used as proxies of U­(VI) specifically bound to carboxyl and phosphoryl ligands in organic matter. A spectrum of dissolved U­(VI) was also considered, accounting for nonspecifically bound species such as in outer-sphere complexes. Figure 4 shows the best fit result of the linear combination analysis with up to three of these standards, indicating that a mix of 66% U-mineral and 34% U-aqueous components (±5%) provides the best reproduction of the experimental data. U­(VI) is known to associate with mineral surface sites in specific inner-sphere complexes,^33–37^ which are captured here in the U-clay fit component. The remaining “aqueous” uranyl component represents the fraction of U­(VI) that is associated with the iron oxides or the supermolecular structure of organic matter through electrostatic interactions, where the hydration sphere of the uranyl remains nearly intact.

**Figure 4:**
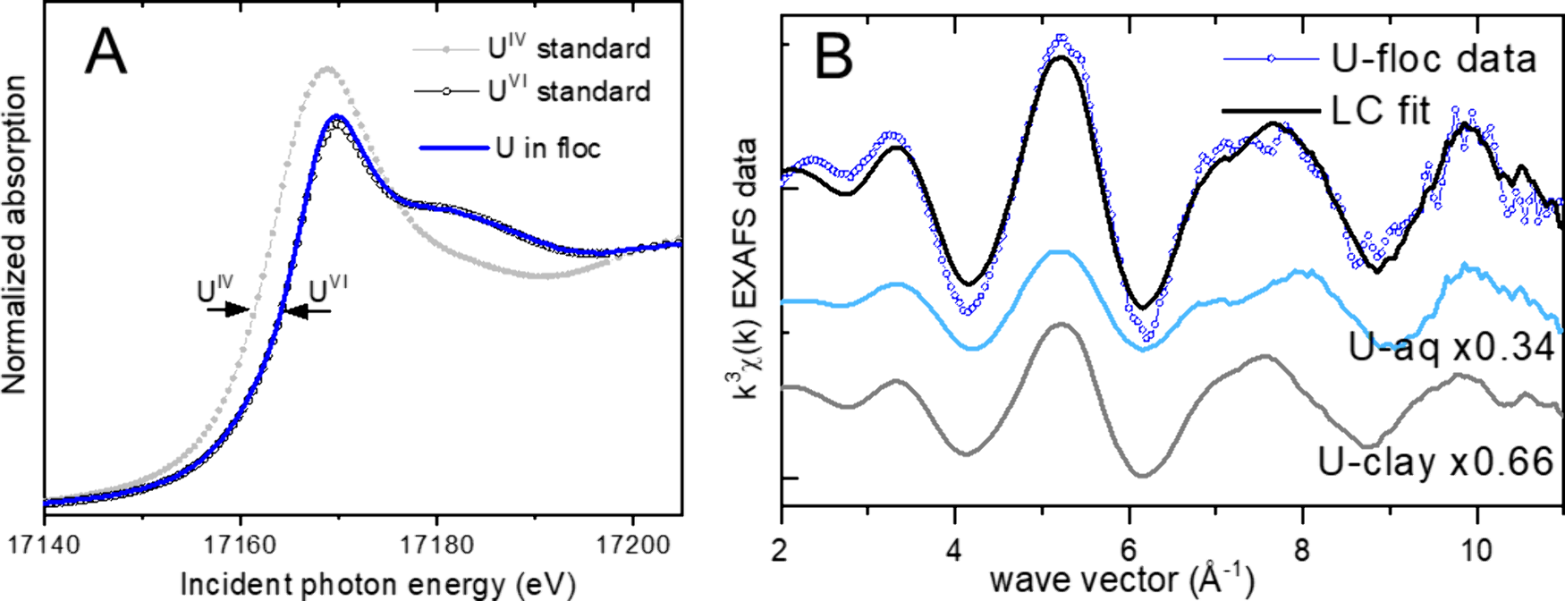
Uranium L_III_-edge X-ray absorption spectra from stream flocs. (A) XANES spectrum compared to standards, indicating the predominance of U­(VI) (100 ± 5%). The standards are U­(VI) adsorbed to SYn-1 clay and U­(IV) in nano-uraninite. (B) Best fit from the linear combination analysis of the EXAFS data. The scaled fit components are offset vertically, and the refined spectral proportions are shown (uncertainties are ±5%). U-clay is the standard of U­(VI) adsorbed to SYn-1 clay, and U-aq is an aqueous solution of U­(VI) representing outer-sphere complexes in the solids.

Together the various measurements made of these stream water samples indicate that the suspended solids during a given sampling event were composed of material originating from two sources: (1) the flocs and (2) the particles resuspended from the stream floor and banks. During the initial stages of a rain event, when the flow rate was still relatively slow, there was a greater contribution of flocs to the stream’s suspended solids load. Once these particles were transported downstream and the flow rate increased, a greater contribution from particles originating from the stream floor and banks comprised the stream’s suspended solids load. The two sources of suspended solids had significantly different potentials for transporting U. The floc-dominated suspended solids had significantly greater U concentrations than those dominated by resuspended solids. An important implication of these findings is that traditional models based on first principles that predict suspended solids stream concentrations based on shearing of particles from the stream floor^2^ would greatly underestimate actual U transport from this wetland system. This underestimation would not only stem from the omission of the U-bearing flocs in the model but also be because the sandy stream bed has been naturally carved 0.2 to 1 m below the contaminated wetland surface, exposing subsurface sediment that commonly contains background levels of U, ∼1 mg/kg U. Data used in the traditional models based on U association with the varying particle size classes of the stream bed sediment would not necessarily provide a good basis for predicting suspended solids U loads. While this second source of suspended solids is somewhat limited to wetlands or perhaps larger gaining streams, the concept that suspended solids have multiple sources may be true of other systems. For example, as streams flood their banks, these flooded lands can release particles with unique contaminant loads or a tendency to remain in suspension. Similarly, particles can be brought into streams via Horton overland flow, and again, such particles could have different contaminant loadings and different tendencies to remain in suspension than stream bed particles. To the best of our knowledge, this is the first study to demonstrate that suspended solids and their ability to transport contaminants can change in a systematic manner during an episodic rain event due to their unique sources.

## Supplementary Material



## Notes

Photographs of the suspended solids and the stream containing flocs; additional figures of rainfall, flow rate, U_<0.45 μm_, and U_>0.45 μm_ for storms B, E, and F; an example of an XRD scan used to generate semiquantitative mineralogy data; linear combination fits to U L_III_-edge spectra from the stream flocs; and U concentrations in suspended solids and flow rate as a function of sampling time (PDF)
