# Supplementary material for: Multiple Sources of Riparian Wetland Suspended Solids during Episodic Rain Events: Influence on Uranium Transport
Source: Environ Sci Technol. 2025 Oct 22;59(43):23475–83. doi: 10.1021/acs.est.5c08896 (PMC12593361; doi:10.1021/acs.est.5c08896)
Supplement: Supplementary file 1 [file es5c08896_si_001.pdf]

Supplemental Information

Multiple sources of riparian wetland suspended solids during episodic rain events: Influence on uranium transport

Daniel I. Kaplan,[a] Karah M. Greene,[a] Wei Xing,[a] Brian A. Powell,[b] Maxim I. Boyanov,[c,d] Edward J. O'Loughlin,[c] Kenneth M. Kemner,[c] Arelis M. Rivera-Giboyeaux,[e] and Peng Lin[a]

[a] Savannah River Ecology Laboratory, University of Georgia, Aiken, South Carolina, 29808, United States
[b] Department of Environmental Engineering & Earth Sciences, Clemson University, Anderson, South Carolina, 29634, United States
[c] Biosciences Division, Argonne National Laboratory, Lemont, Illinois, 60439, United States
[d] Institute of Chemical Engineering, Bulgarian Academy of Sciences, Sofia, 1113, Bulgaria
[e] Atmospheric Technologies Group, Savannah River National Laboratory, Aiken, South Carolina, 29808, United States

*This supplemental information contains **6** pages*

List of Figures

Figure S1. Top: Suspended solids on 0.45-μm filters collected from time series sampling during sampling event E. Bottom Left: Stream during period when naturally occurring floc concentrations were high. Bottom Right: Stream a few days after storm event had washed flocs downstream.

Figure S2. Rainfall, flow rate, $U_{<0.45\mu m}$, and $U_{>0.45\mu m}$, during sampling events B, E, and F. Similar plots for sampling events A and D are presented in the manuscript (Figure 2).

Figure S3. Example of XRD scan used to generate the semi-quantitative XRD data presented in Table 4. Sample is a composite of suspended solids from late samples (collected after the peak flow rate) from event E. A silicon spike was mixed into the sample to enable quantification estimates.

Figure S4. Linear combination fits of the U L_{III}-edge spectra from the stream flocs. The scaled fit components are offset vertically. Refined proportions are shown next to each component (uncertainties are ±5%).

Figure S5. Uranium concentrations in suspended solids and flow rate as a function of sampling time for each of the five sampling events.

Figure S1. Top: Suspended solids on 0.45-μm filters collected from time series sampling during sampling event E. **Bottom Left:** Stream during period when naturally occurring floc concentrations were high. **Bottom Right:** Stream a few days after storm event had washed flocs downstream.

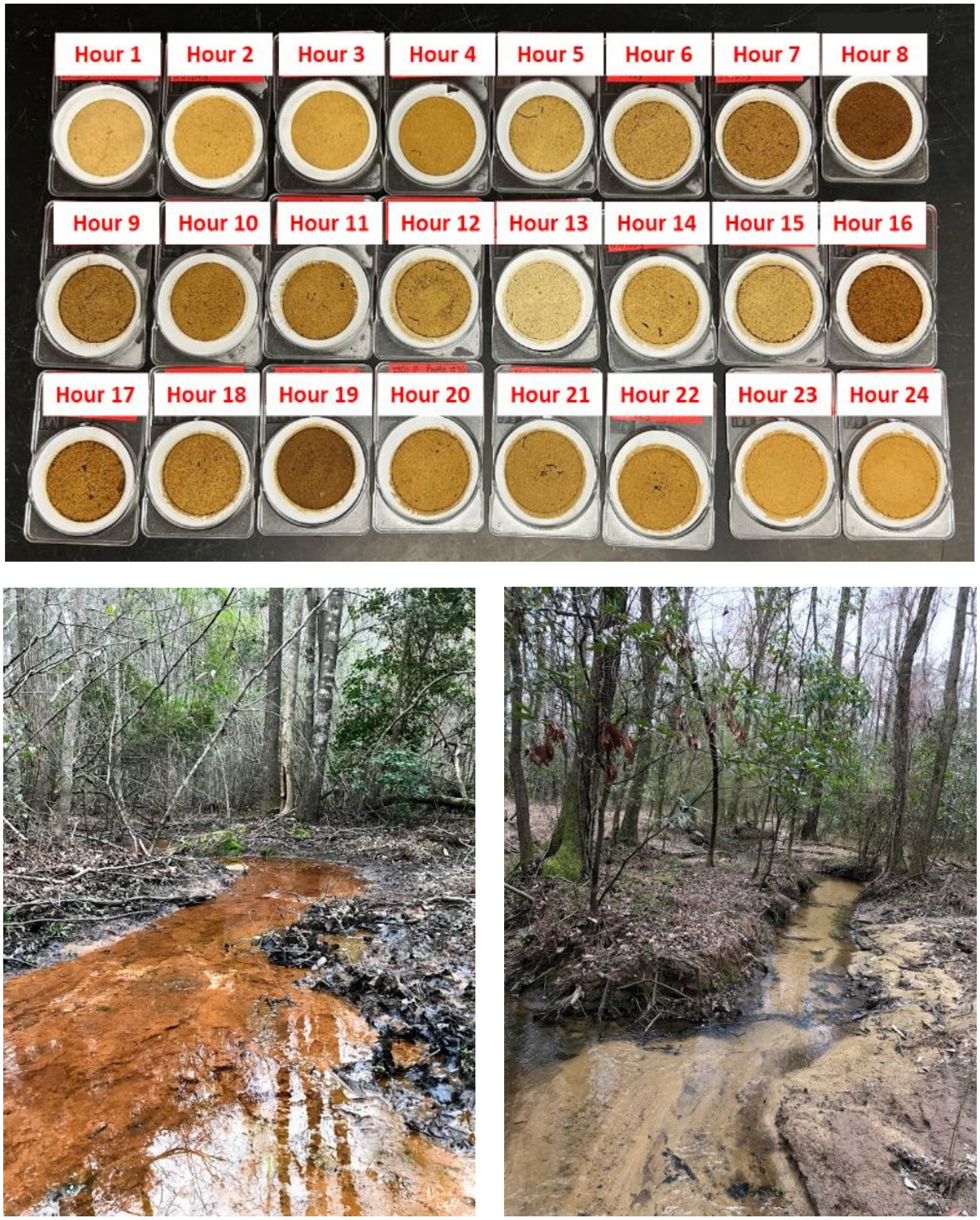

Figure S2. Rainfall, flow rate, $U_{<0.45\mu m}$, and $U_{>0.45\mu m}$, during sampling events B, E, and F. Similar plots for Sampling Events A and D are presented in manuscript (Figure 2).

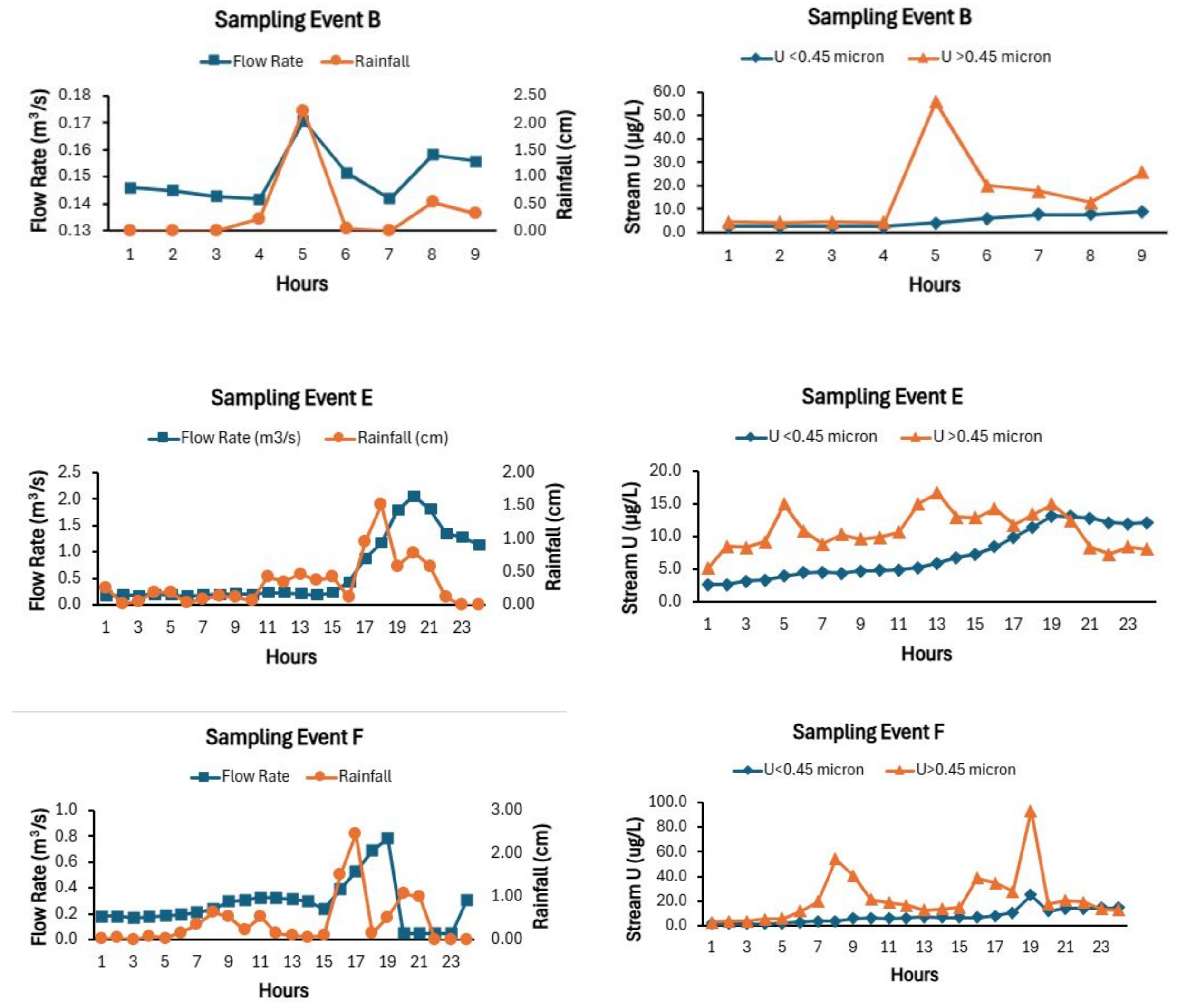

Figure S3. Example of XRD scan used to generate the semi-quantitative XRD data presented in Table 4. Sample is a composite of suspended solids from late samples (collected after the peak flow rate) from event E. A silicon spike was mixed into the sample to enable quantification estimates.

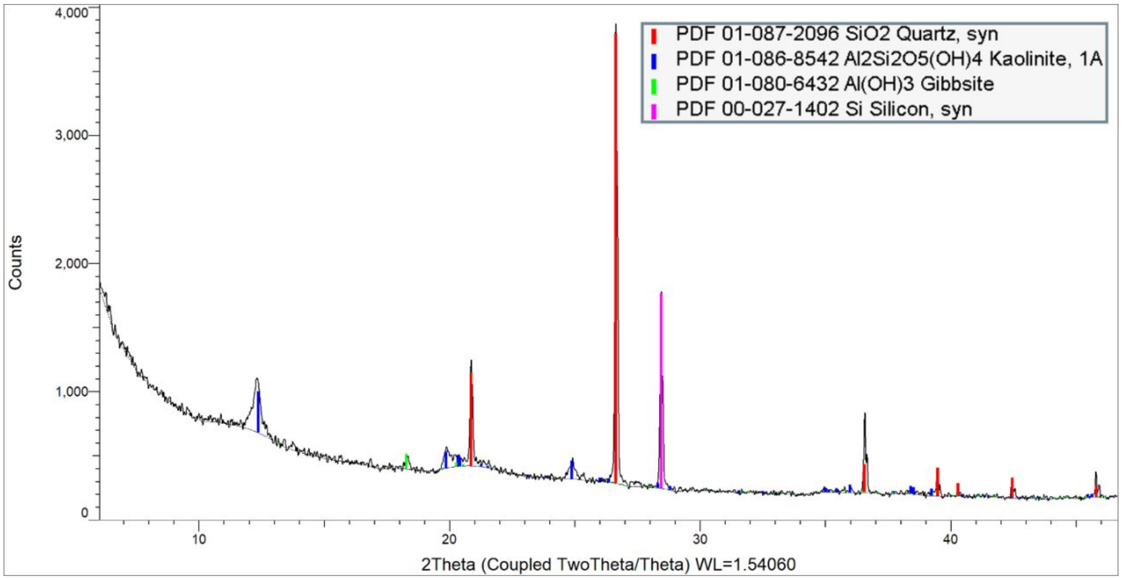

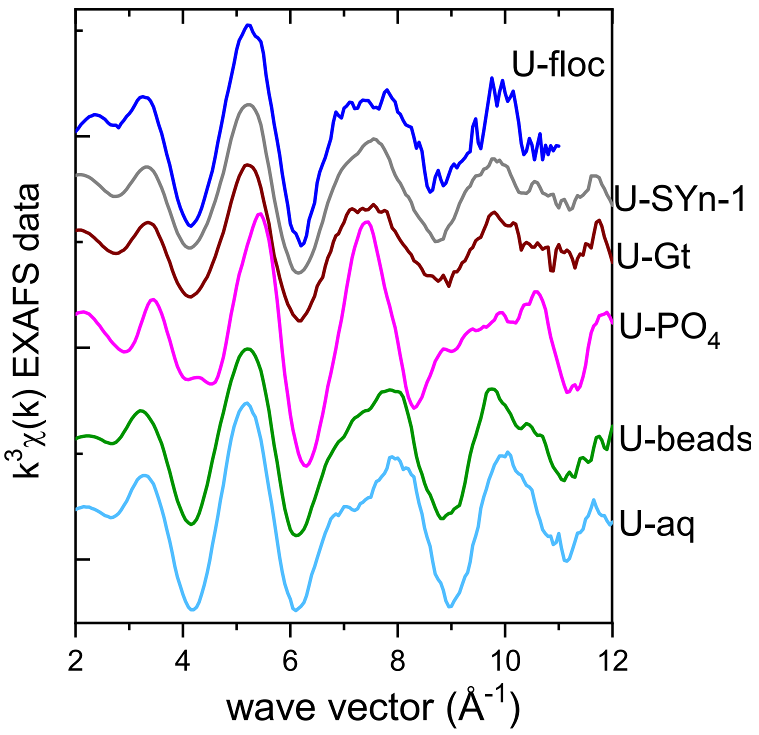

Figure S4. Linear combination fits of the U L_{III}-edge spectra from the stream flocs. The scaled fit components are offset vertically. Refined proportions are shown next to each component (uncertainties are ±5%).

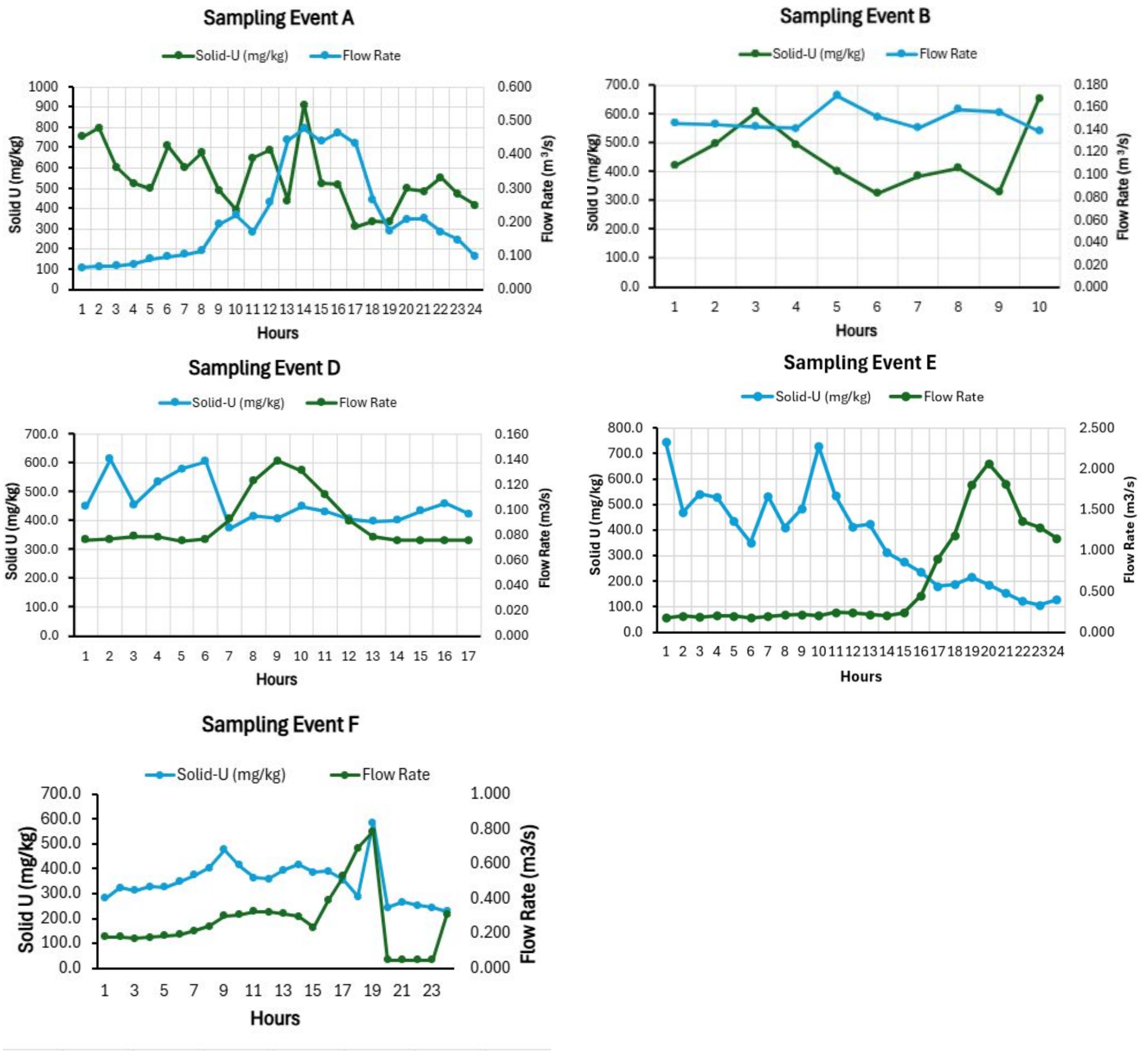

Figure S5. Uranium concentrations in suspended solids and flow rate as a function of sampling time for each of the five sampling events.
